# ON or OFF?: Modulating the N-Methyl-D-Aspartate Receptor in Major Depression

**DOI:** 10.3389/fnmol.2016.00169

**Published:** 2017-01-13

**Authors:** Shi Yu Chan, Edward Matthews, Philip W. J. Burnet

**Affiliations:** ^1^Department of Psychiatry, Warneford Hospital, University of OxfordOxford, UK; ^2^Green Templeton College, University of OxfordOxford, UK

**Keywords:** NMDAR antagonist, glycine site, mTOR, depression, subunit

## Abstract

Since the discovery that a single dose of ketamine, an N-methyl-D-aspartate receptor (NMDAR) antagonist, had rapid and long-lasting antidepressant effects, there has been increased interest in using NMDAR modulators in the pharmacotherapy of depression. Ketamine’s efficacy seems to imply that depression is a disorder of NMDAR hyperfunctionality. However, studies showing that not all NMDAR antagonists are able to act as antidepressants challenge this notion. Furthermore, NMDAR co-agonists have also been gaining attention as possible treatments. Co-agonists such as D-serine and sarcosine have shown efficacy in both pre-clinical models and human trials. This raises the question of how both NMDAR antagonists and agonists are able to have converging behavioral effects. Here we critically review the evidence and proposed therapeutic mechanisms for both NMDAR antagonists and agonists, and collate several theories on how both activation and inhibition of NMDARs appear to have antidepressant effects.

## Introduction

The N-methyl-D-aspartate receptors (NMDARs) are a class of ionotropic glutamate receptors that are widely expressed in the brain. They are composed of two glycine-binding GluN1 subunits and two glutamate-binding GluN2 subunits (GluN2A, GluN2B, GluN2C and GluN2D). In the adult brain, the majority of NMDARs are a combination of GluN1 with GluN2A and/or GluN2B (Papadia and Hardingham, 2007), that play important roles in neurodevelopment, synaptic plasticity, learning and memory (Morris et al., 1986; Riedel et al., 2003; Hunt and Castillo, 2012; Burnashev and Szepetowski, 2015). Conversely, dysregulation of NMDARs is associated with some neuropsychiatric disorders, such as schizophrenia, where NMDAR hypofunction has been evinced through the psychotomimetic effects of NMDAR antagonists (Olney et al., 1999), and NMDAR hyperfunction has been associated with excitotoxicity and neurodegeneration (Zhou et al., 2013). This has led to the inverted-U curve hypothesis of NMDAR function (Lipton and Nakanishi, 1999), and highlighted NMDAR modulators as potential therapeutic interventions for neuropsychiatric disorders.

The NMDAR co-agonists, D-serine, D-alanine and glycine, and glycine uptake inhibitors, have proved effective at ameliorating negative symptoms of schizophrenia when used as adjunctive therapies (Heresco-Levy et al., 2004, 2005; Tsai et al., 2004, 2006; Kantrowitz et al., 2010), and support the NMDAR hypofunction theory for this disorder. The NMDAR antagonist, memantine, has proved to be therapeutically beneficial in some cases of Alzheimer’s disease (Reisberg et al., 2003), where glutamate-mediated neuropathology is posited. However, recent attention has focused on the NMDAR as a therapeutic target for major depression, and despite often ambiguous mechanistic insight, both inhibition and stimulation of this receptor convey antidepressant properties. This review article will critically evaluate the current literature reporting the validity of NMDAR modulation in major depression, and will propose a mechanism by which the function of this receptor in an “on” or “off” state may have antidepressant actions.

## NMDAR Modulation as a Therapeutic Strategy: Conflicting Evidence

**Figure 1:**
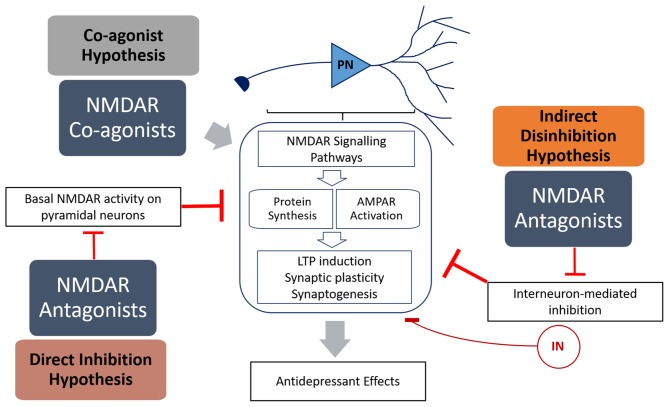
**Summary of the mechanisms of how N-methyl-D-aspartate receptor (NMDAR) antagonists (direct inhibition and disinhibition) and co-agonists lead to antidepressant effects.** The indirect hypothesis proposes that NMDAR antagonists inhibit the basal activation of inhibitory interneurons, resulting in disinhibition of pyramidal neurons. The direct hypothesis proposes that NMDAR antagonists inhibit basal activation of pyramidal neurons (caused by spontaneous or ambient glutamate) that in turn inhibits protein synthesis. The co-agonist hypothesis proposes that NMDAR co-agonists activate signaling pathways in pyramidal neurons that result in increased synaptic plasticity. Both NMDAR antagonists and agonists activate signaling pathways that result in increased protein translation and α-amino-3-hydroxy-5-methyl-4-isoxazolepropionic acid receptor (AMPAR) activation, leading to increased LTP induction, synaptic plasticity and antidepressant behavior.

Interest in the utility of NMDAR modulators in depression developed when a single sub-anesthetic dose of ketamine, a non-competitive NMDAR antagonist, was shown to produce rapid and long-lasting antidepressant effects (Berman et al., 2000). However, while much headway has been made in elucidating the mechanisms behind ketamine’s efficacy, our understanding of the role of NMDARs in mood disorders is far from complete. Added to this is the complexity of the different sub-environments of different brain regions, different types of neurons (i.e., pyramidal neurons and interneurons) and the diversity of NMDAR subunits and regulators. Given the volume of information obtained from research on ketamine, it appears that NMDAR antagonists have great potential as a new class of antidepressants. This is supported by studies on other NMDAR antagonists, such as nitrous oxide (Zorumski et al., 2015) and lanicemine (Sanacora et al., 2014; Downey et al., 2016), which show great promise as potential antidepressants in pre-clinical models. However, memantine does not display antidepressant properties (Zarate et al., 2006), and numerous NMDAR agonists, in particular agonists of the glycine site (e.g., GLYX-13, Moskal et al., 2014), may be potential treatments for depression. This raises the question of how both NMDAR antagonists and agonists are able to have antidepressant effects (Figure 1).

## NMDAR Antagonists: The Mechanism of Ketamine

Ketamine is an anesthetic and a psychotomimetic drug (Krystal et al., 1994) with antidepressant properties (Berman et al., 2000). Recently, Miller et al. (2016) reviewed the evidence behind two dominant hypotheses explaining ketamine’s mode of action—direct inhibition, and disinhibition (Figure 1). The “disinhibition” theory proposes that ketamine antagonizes NMDARs on inhibitory interneurons, therefore removing the inhibition of pyramidal neurons, and increasing glutamate neurotransmission. The “direct inhibition” theory, however, proposes that NMDARs are tonically activated by ambient glutamate and glutamate from spontaneous-releasing synaptic vesicles, and that this detrimental tonic activation is directly inhibited by ketamine.

Although a consensus has not been reached for the initiation of ketamine’s activity, research has highlighted certain key elements that may provide a clue for how NMDAR antagonists work as antidepressants. Research has shown that ketamine blockade of NMDARs inhibits the eukaryotic elongation factor 2 (eEF2) kinase, which leads to eEF2 de-phosphorylation and de-suppression of Brain-Derived-Neurotrophic-Factor (BDNF) translation (Autry et al., 2011). The levels of BDNF and its receptor, Tropomyosin receptor kinase B (TrkB), have been positively correlated with antidepressant efficacy, possibly through their roles in synaptogenesis and neurogenesis (Saarelainen et al., 2003; Duman and Monteggia, 2006; Leal et al., 2014). BDNF has also been shown to activate the mammalian target of rapamycin (mTOR) signaling pathway (Nosyreva et al., 2013), which is involved in protein synthesis and increased excitatory neurotransmission (Miller et al., 2014).

Studies with NBQX, an α-amino-3-hydroxy-5-methyl-4-isoxazolepropionic acid receptor (AMPAR) antagonist, and rapamycin, an mTOR inhibitor, have also shown that the AMPARs and the mTOR signaling pathway are essential for ketamine’s antidepressant effects. Li et al. (2010) have shown that ketamine activated growth factor signaling proteins, increased levels of synaptic proteins and AMPAR subunits, and increased dendritic spine densities. These effects were abolished by inhibitors of mTOR, extracellular signal-regulated kinases (ERK) and Protein kinase B (Akt).

Intuitively, all NMDAR non-competitive antagonists might be expected to function in a similar way to ketamine, but that does not hold true for memantine. One explanation is that memantine, unlike ketamine, is a poor blocker of resting NMDAR currents, and that ketamine is a “resting NMDAR blocker”, which supports the “direct inhibition” theory of ketamine’s antidepressant action (Gideons et al., 2014; Kavalali and Monteggia, 2015). Moreover, memantine neither induced eEF2 de-phosphorylation nor increased BDNF expression (Gideons et al., 2014), and its activity is not affected by mTOR inhibition (Sabino et al., 2013). Another theory for these differential effects of memantine and ketamine is that they could be binding to distinct NMDAR “subpopulations” (Johnson et al., 2015), which might include NMDAR subunit composition and/or synaptic location, though this remains controversial (Wroge et al., 2012; Emnett et al., 2013; Gideons et al., 2014).

## NMDAR Agonists: Molecular Mechanisms

Given the evidence supporting NMDAR antagonists as a new class of antidepressants, it is counter-intuitive that NMDAR agonists are also able to act as antidepressants. In particular, co-agonists of the GluN1 subunit (GLYX13, D-serine) have been shown to improve mood in healthy volunteers, and reduce indices of behavioral despair in rodent behavioral tasks such as the forced-swim test (FST) and the learned helplessness (LH) paradigm (Malkesman et al., 2012; Burgdorf et al., 2013; Levin et al., 2015). In the FST, animals administered with the NMDAR co-agonists exhibited reduced immobility (despondency) in water, which indicated an antidepressant-like effect. In the LH task, the latency to escape a foot-shock is a measure of depressive-like behavior, and NMDAR co-agonists reduced this parameter. Burgdorf et al. (2013) also demonstrated that GLYX13 and ketamine increased surface GluN2B and GluR1 levels in the medial prefrontal cortex (mPFC) and the hippocampus, and increased excitatory post-synaptic current (EPSC) in the hippocampus. This suggested that, in spite of their differential pharmacological properties, common molecular pathways are activated by both compounds.

The glycine transporter inhibitor, sarcosine, has also been shown to attenuate immobility in the FST in mice, and improve mood scores in depressed patients (Huang et al., 2013; Mathew, 2013). An investigation into its psychotropic mechanisms revealed that it induced the phosphorylation of mTOR, ERK and Akt, as well as GluR1 which implies increased membrane insertion of AMPARs (Chen et al., 2015). Parenthetically, although sarcosine is generally recognized as a glycine transporter inhibitor, it also possesses NMDAR co-agonist properties, and can enhance the activation of this receptor through its direct binding to the glycine site on the GluN1 subunit (Zhang et al., 2009a). The antidepressant effects of sarcosine can, therefore, be attributed to the inhibition of synaptic glycine uptake, and/or direct NMDAR stimulation. Nevertheless, overall it appears that mTOR signaling and AMPARs are common downstream targets of both NMDAR agonists and antagonists. This raises the question of how NMDAR modulators that have opposing effects on NMDARs could lead to similar downstream effects. Several theories that have been proposed to explain this phenomenon will now be considered.

## Partial Agonists as Potential Antagonists: Dual Effects of GluN1 Agonists

Many GluN1 co-agonists are partial agonists that may act as antagonists at higher concentrations, and the most extensively studied of these is D-cycloserine (DCS). It has been proposed that at low concentrations and when the NMDAR glycine site is not fully occupied, DCS acts as an agonist, albeit with a lower efficacy than glycine (van Berckel et al., 1999). At higher concentrations however, DCS can compete with, and block, glycine’s co-agonist function, thereby reducing activity of NMDARs. This is supported by pre-clinical models and clinical trials for depression and schizophrenia that show the contrasting dose-dependent effects of DCS. While DCS has been shown to improve negative symptoms of schizophrenia in humans at doses below 250 mg, doses above that exacerbate the positive symptoms (van Berckel et al., 1999). The reverse is true for depression where doses above 250 mg are necessary to observe antidepressant effects in an add-on clinical trial (Heresco-Levy et al., 2013), suggesting that DCS’s antidepressant effects are achieved when it acts as an NMDAR antagonist.

## AMPAR Convergence

Current literature supports the notion that AMPARs may play an important role in the efficacy of antidepressants. For example, chronic administration of paroxetine and fluoxetine increase the total (Martinez-Turrillas et al., 2002) and phosphorylated levels of GluR1 (Svenningsson et al., 2002, 2007). The importance of AMPAR potentiation has also been demonstrated in studies using positive modulators of AMPARs, such as LY451646 (Andreasen et al., 2015), and in investigations using AMPAR antagonists where the antidepressant properties of fluoxetine and ketamine are blocked (Farley et al., 2010; Li et al., 2010). More recently, Zanos et al. (2016) attributed the antidepressant effects of ketamine to the action of ketamine metabolites on AMPARs.

The activity of AMPAR has also been shown to be important for synaptic potentiation, the mechanism thought to underlie antidepressant actions. While ketamine increased total field potential in the hippocampus, NMDAR-specific field potential decreased, implying that AMPAR and other non-NMDARs contributed to the increase (Nosyreva et al., 2013). This has led to the theory that the key mechanism of action of antidepressants is the increased ratio of AMPAR/NMDAR activity rather than NMDAR antagonism (Andreasen et al., 2013). Therefore, increasing AMPAR activity, decreasing NMDAR activity, or both, would achieve an antidepressant effect. The NMDAR antagonists, such as ketamine, that inhibit NMDAR activity would lead to an increased AMPAR/NMDAR activity ratio. However, drugs that are able to increase AMPAR activity through an NMDAR-independent pathway would also lead to an increased AMPAR/NMDAR activity ratio. For instance, glycine treatment has been shown to increase AMPAR insertion in the synaptic membrane (Lu et al., 2001), which may surpass its NMDAR stimulatory properties. Furthermore, Andreasen et al. have demonstrated that a positive AMPAR modulator and NMDAR antagonist have synergistic antidepressant effects in mice (Andreasen et al., 2013).

## Non-NMDAR Targets

When exploring the mechanisms of NMDAR modulators, the assumption made is that the key mode of action of these drugs are NMDAR-dependent. However, this may not be the case. For example, glycine has a high affinity for post-synaptic NMDARs at low concentrations (Zhang et al., 2014), but greater levels in the synaptic cleft could result in spill-over and additional binding to extra-synaptic inhibitory strychnine-sensitive glycine receptors (GlyRs). Thus, elevated synaptic glycine has been associated with increased NMDAR EPSCs and LTP (Johnson and Ascher, 1987), whereas exogenously applied glycine appears to have an inhibitory effect on NMDARs, and subsequent LTD (Chen et al., 2011). Therefore, the same co-agonist could lead to either LTP or LTD, depending on which receptor it preferentially activates (Zhang et al., 2014). Sarcosine may have potentially three modes of action since it is a glycine transport inhibitor (Smith et al., 1992), a NMDAR co-agonist, and has some GlyR agonist properties (Zhang et al., 2009b). Ketamine itself has been shown to bind to and affect non-glutamatergic pathways (Sleigh et al., 2014), such as the opioid system (Gupta et al., 2011). Indeed, it has also been shown that administration of ketamine metabolites result in antidepressant behavior independent of NMDAR inhibition, but dependent on AMPAR activation (Zanos et al., 2016).

## “Full” Co-Agonists

Arguably, the antidepressant action of partial agonists, such as DCS, might be explained by their potential antagonist properties, which is consistent with non-competitive antagonists such as ketamine having the same behavioral effects. However, this does not account for the actions of D-serine, an endogenous co-agonist of the GluN1 subunit that does not act as an antagonist at high doses (Kleckner and Dingledine, 1988; Berger et al., 1998; Mothet et al., 2000). Unlike DCS, sarcosine, and GLYX13, D-serine has a higher affinity than glycine for the NMDAR glycine site and does not bind to other targets (Matsui et al., 1995; Levin et al., 2015). Nevertheless, D-serine has been shown to have antidepressant effects.

An acute dose of D-serine reduced feelings of anxiety and sadness and improved cognitive scores in healthy volunteers (Levin et al., 2015). Likewise, in rodents, an acute dose of D-serine led to improvements in the FST, female urine sniffing test (FUST), and LH paradigm, similar to the antidepressant effects observed after a single dose of ketamine (Malkesman et al., 2012). These actions of D-serine have also been shown to be occluded in GluN1-knock out mice. Reduced immobility in the FST, and reduced latency in the novelty-suppressed feeding test were also observed in mice chronically fed with D-serine, or when the D-serine synthesizing enzyme, serine racemase, was over-expressed (Otte et al., 2013). So how can the antidepressant properties of both D-serine and ketamine be explained? Could preference for particular NMDAR-subtypes, their extra-synaptic or synaptic expression, and/or their neuroanatomical location influence the action of receptor modulators?

## NMDAR Subtype Specificity and Localization

Given that NMDARs are composed of different subunits, some emphasis has been placed on the differential physiological properties of receptors composed of different subunit subtypes, in particular GluN2A and GluN2B. Various groups have shown that the GluN2A and GluN2B subtypes are functionally distinct. A popular theory is that GluN2B-containing NMDARs are linked to neurodegeneration, whilst GluN2A-containing NMDARs are linked to neuroprotection (Lujan et al., 2012). Also, different NMDAR subtypes are dominant at different stages of development (Wenzel et al., 1997). For instance, the GluN2B-containing NMDARs play a key role in cortical development, and their loss of function, achieved by knocking out GluN2B, cannot be rescued with other subunits, such as GluN2A (Wang et al., 2011). Furthermore, GluN2B activation has been linked to the suppression of protein synthesis and decreased miniature EPSCs in both developing and adult rodents (Wang et al., 2011; Miller et al., 2014). The subtypes also have different characteristics. The GluN2B subunit, compared to GluN2A, displays higher sensitivity to agonists, and differential sensitivity to the magnesium block (Kuner and Schoepfer, 1996; Erreger et al., 2007; Miller et al., 2014). However, this raises the question of how subtype differences can result in different downstream effects. Several mechanisms have been proposed.

First, the synaptic localization of different subtypes could explain differences in function. That is, GluN2A-containing NMDARs are primarily expressed at synaptic sites, while GluN2B-containing NMDARs are mostly expressed at extra-synaptic sites (Massey et al., 2004). Second, the GluN2 subtypes may convey their specific effects through their structural differences. Experiments have shown GluN2A and GluN2B might regulate different signaling cascades through variances in their intracellular C-terminal tail (Foster et al., 2010). At the ligand binding site, several NMDAR modulators and potential therapeutics have shown a preference for a particular GluN2 subtype (Williams, 1993; Fischer et al., 1997; Paoletti et al., 1997; Nozaki et al., 2011). Interestingly, the GluN2B-containing NMDARs are proposed to underlie the effects of ketamine and GLYX13, because their actions are blocked by GluN2B-specific antagonists, and both compounds increase cell surface levels of GluN2B (Burgdorf et al., 2013). Perhaps more pertinent is the demonstration that the antidepressant fluoxetine binds directly to, and blocks, GluN2B-containing NMDARs (Szasz et al., 2007; Kiss et al., 2012).

Finally, Miller et al. (2014) have shown that GluN2B-containing NMDARs are more sensitive to ambient and spontaneous glutamate release, proposing a mechanism where ketamine’s direct inhibition of tonic ambient glutamate neurotransmission results in disinhibition of protein synthesis and an overall increase in AMPAR activation and synaptic signaling. Given that Papouin et al. (2012) have demonstrated that synaptic and extra-synaptic NMDARs are gated by different endogenous co-agonists, D-serine and glycine respectively, and that glycine-gated extra-synaptic NMDARs control the ambient glutamate-generated tonic current, it suggests a mechanism where ketamine preferentially inhibits an extra-synaptic pool of NMDARs mainly composed of a GluN2B subunit.

**Figure 2:**
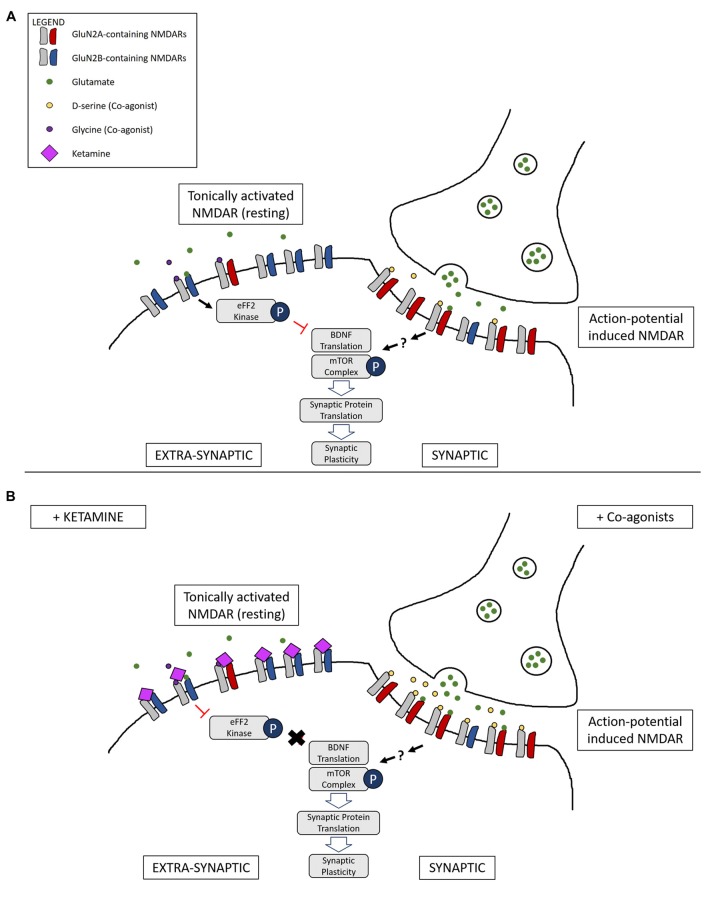
**Illustration of a complementary mechanism providing an explanation for how both NMDAR co-agonists and antagonists are able to lead to similar downstream antidepressant effects. (A)** Differential activation of NMDARs based on location: tonic activation of extra-synaptic NMDARs by ambient/spontaneous glutamate release lead to inactivation of the mammalian target of rapamycin (mTOR) signaling complex and the inhibition of protein synthesis. On the other hand, action-potential stimulated release of glutamate and the presence of the synaptic co-agonist D-serine leads to activation of synaptic NMDARs, triggering pathways that lead to protein synthesis and synaptic plasticity. **(B)** Main proposed mechanism underlying complementary antidepressant effects of NMDAR antagonists and agonists: NMDAR antagonists, such as ketamine, inhibit the tonic activation of extra-synaptic NMDARs, resulting in activation of the mTOR signaling complex and protein synthesis. Administration of NMDAR co-agonists, such as D-serine, occupy un-saturated glycine sites on NMDARs, stimulating synaptic NMDARs and leading to LTP and antidepressant effects. Ketamine binding at synaptic sites is not represented.

One model, therefore, is that NMDAR agonists preferentially stimulate synaptic NMDARs (action-potential-driven neurotransmission), while NMDAR antagonists block extra-synaptic NMDARs (spontaneous or ambient glutamate-driven neurotransmission; Figure 2). However, research on this has been controversial and a consensus on the exact roles and locations of NMDARs composed of specific subtypes have yet to be elucidated. It has even been suggested that extra-synaptic NMDARs may exist as separate pools with different modes of activation and functions (Papouin and Oliet, 2014).

## Conclusion

The modulation of the NMDAR as a potential therapeutic strategy for major depression is supported by compelling evidence, though existing clinical data have not yet eluded to a pathophysiological role of NMDARs in this disorder. That is, both NMDAR antagonists (e.g., ketamine) and agonists (e.g., D-serine) have therapeutic actions in depression, unlike in schizophrenia where the exacerbation of symptoms by ketamine, and their improvement by D-serine, suggests NMDAR hypofunction in psychotic illness. However, molecular considerations do argue the involvement of NMDAR dysfunction in the pathogenesis of depression, particularly with respect to ambient glutamate-driven neurotransmission. Receptor antagonists block this detrimental tonic activation of extra-synaptic NMDARs, while agonists promote synaptic glutamate neurotransmission that leads to synaptic potentiation (Figure 2). In this regard, the therapeutic actions of both receptor antagonists and agonists are accommodated. Of course, further investigations are required to support or refute this hypothesis. Additional patient studies are also required both to substantiate the antidepressant actions of NMDAR modulators, and to show if depressed subjects can be stratified into agonist and/or antagonist responders and non-responders. Nevertheless, molecular studies propose that both NMDAR inhibition and stimulation converge on the activation of BDNF/mTOR signaling. Therefore, it would be interesting to test whether a combination of GluN1 agonist and NMDAR antagonist would have synergistic, reinforcing effects.

## Author Contributions

SYC, EM and PWJB made a substantial contribution to the conception, writing and structure of the review article. EM prepared the first draft of the manuscript, and SYC provided subsequent drafts and the final article. PWJB supervised and proof read the manuscript.

## Funding

SYC is supported by the National Science Scholarship (NSS) from the Agency of Science, Technology and Research (A*STAR) in Singapore. PWJB currently receives research funding from the Biotechnology, Biological Sciences Research Council, UK (BBSRC, BB/N010035/1).

## Conflict of Interest Statement

The authors declare that the research was conducted in the absence of any commercial or financial relationships that could be construed as a potential conflict of interest.
